# Medulloblastoma Associated with Down Syndrome: From a Rare Event Leading to a Pathogenic Hypothesis

**DOI:** 10.3390/diagnostics11020254

**Published:** 2021-02-07

**Authors:** Alessandra Boni, Marco Ranalli, Giada Del Baldo, Roberto Carta, Mariachiara Lodi, Emanuele Agolini, Martina Rinelli, Diletta Valentini, Sabrina Rossi, Viola Alesi, Antonella Cacchione, Evelina Miele, Iside Alessi, Anna Maria Caroleo, Giovanna Stefania Colafati, Maria Antonietta De Ioris, Luigi Boccuto, Mario Balducci, Andrea Carai, Angela Mastronuzzi

**Affiliations:** 1 Department of Pediatrics, Sapienza University, Viale Regina Elena 324, 00161 Rome, Italy; lale.boni88@gmail.com (A.B.); ranalli.marco91@gmail.com (M.R.); 2 Department of Onco-Hematology and Cell and Gene Therapy, Bambino Gesù Children’s Hospital IRCCS, Piazza Sant’Onofrio 4, 00146 Rome, Italy; giada.delbaldo@opbg.net (G.D.B.); roberto.carta@opbg.net (R.C.); mariachiara.lodi@opbg.net (M.L.); antonella.cacchione@opbg.net (A.C.); evelina.miele@opbg.net (E.M.); iside.alessi@opbg.net (I.A.); annamaria.caroleo@opbg.net (A.M.C.); mantonietta.deioris@opbg.net (M.A.D.I.); 3 Laboratory of Medical Genetics, IRCCS Bambino Gesù Children’s Hospital, Piazza Sant’Onofrio 4, 00146 Rome, Italy; emanuele.agolini@opbg.net (E.A.); martina.rinelli@opbg.net (M.R.); viola.alesi@opbg.net (V.A.); 4 Pediatric and Infectious Disease Unit, Bambino Gesù Children’s Hospital, IRCCS, Piazza Sant’Onofrio 4, 00146 Rome, Italy; diletta.valentini@opbg.net; 5 Department of Laboratories, Pathology Unit, Bambino Gesù Children’s Hospital, Piazza Sant’Onofrio 4, 00146 Rome, Italy; sabrina.rossi@opbg.net; 6 Neuroradiology Unit, Department of Imaging, Bambino Gesù Children’s Hospital, IRCCS, Piazza Sant’Onofrio 4, 00146 Rome, Italy; gstefania.colafati@opbg.net; 7 School of Nursing, College of Behavioral, Social and Health Sciences, Clemson University, Clemson, SC 29634, USA; lboccuto@clemson.edu; 8 JC Self Research Institute of the Greenwood Genetic Center, Greenwood, SC 29646, USA; 9 Department of Imaging, Radiation Oncology and Haematology, Policlinico A. Gemelli Fundation, IRCCS, Catholic University of Sacred Heart, Largo A. Gemelli 1, 00168 Rome, Italy; mario.balducci@policlinicogemelli.it; 10 Neurosurgery Unit, Department of Neurological and Psychiatric Sciences, Bambino Gesù Children’s Hospital, IRCCS, Piazza Sant’Onofrio 4, 00146 Rome, Italy; andrea.carai@opbg.net

**Keywords:** brain tumor, Down syndrome, medulloblastoma, cancer predisposition syndrome

## Abstract

Down syndrome (DS) is the most common chromosome abnormality with a unique cancer predisposition syndrome pattern: a higher risk to develop acute leukemia and a lower incidence of solid tumors. In particular, brain tumors are rarely reported in the DS population, and biological behavior and natural history are not well described and identified. We report a case of a 10-year-old child with DS who presented with a medulloblastoma (MB). Histological examination revealed a classic MB with focal anaplasia and the molecular profile showed the presence of a *CTNNB1* variant associated with the wingless (WNT) molecular subgroup with a good prognosis in contrast to our case report that has shown an early metastatic relapse. The nearly seven-fold decreased risk of MB in children with DS suggests the presence of protective biological mechanisms. The cerebellum hypoplasia and the reduced volume of cerebellar granule neuron progenitor cells seem to be a possible favorable condition to prevent MB development via inhibition of neuroectodermal differentiation. Moreover, the NOTCH/WNT dysregulation in DS, which is probably associated with an increased risk of leukemia, suggests a pivotal role of this pathway alteration in the pathogenesis of MB; therefore, this condition should be further investigated in future studies by molecular characterizations.

## 1. Introduction

Down syndrome (DS), also known as trisomy 21, is the most common chromosomal abnormality among live births, with an incidence varying between 1:400 to 1:3000 live births, presenting a wide spectrum of clinical features, including dysmorphic traits, cardiac, gastrointestinal abnormalities, cognitive impairment, hearing loss, and immune and endocrine deficiencies [1]. DS is a cancer predisposition syndrome, with a 10- to 20-fold higher risk of developing leukemia (particularly myeloid type) compared to the general population and a lower risk for solid tumors, exhibiting a unique pattern of malignancies [2,3]. These particular findings in people with DS may provide clues in the search for leukemogenic genes and tumor suppressor genes on chromosome 21. However, the molecular mechanisms leading to cancer predisposition in the presence of this trisomy are not fully understood.

Medulloblastoma (MB) is the most common malignant tumor of the central nervous system (CNS) in children, representing 9.2% of pediatric CNS tumors [4]. Fewer than 5% of MB cases are associated with hereditary cancer predisposition syndromes (CPSs) [5,6,7,8], and MB cases are rarely described in the DS population [9,10].

To our knowledge, this report presents the first case of a MB with wingless subgroup (WNT) in an individual with DS. We reviewed the molecular characterizations of MB in DS patients reported in the literature and formulated hypotheses on the low incidence of MB in DS. 

## 2. Case Presentation

**Figure 1:**
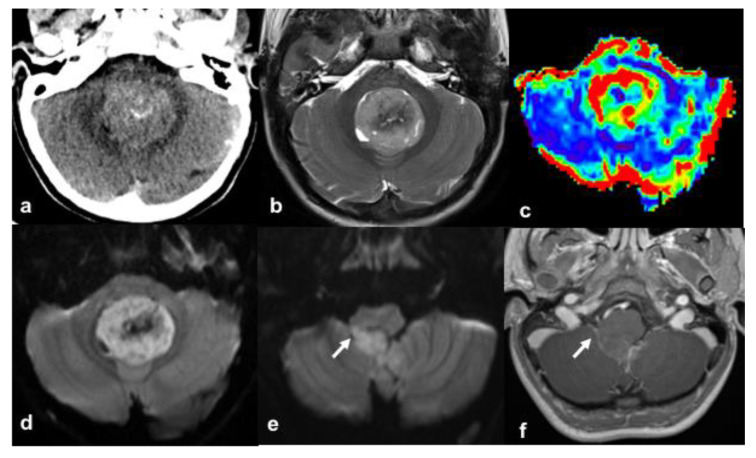
Axial computed tomography (CT) (**a**) and magnetic resonance imaging (MRI) (T2w (**b**); arterial spin labeling (**c**); diffusion weighted imaging (**d**,**e**); GdT1 (**f**)) axial images. CT study shows a fourth ventricle mass with lesional mineralizations (**a**). The tumor has a heterogeneous signal on MRI (**b**), increased perfusion (**c**), restriction of the diffusivity (**d**), and mild contrast enhancement (**f**). Furthermore, the involvement of the right foramen of Luschka is noted (arrow, (**e**,**f**)).

A 10-year-old Caucasian boy, born after medically assisted conception (in vitro fertilization) with DS presenting with congenital hypothyroidism (CH), cognitive impairment, and bilateral transmission hearing loss, came to our attention after about two weeks of daily vomiting, fatigue, headache, and progressive appearance of walking instability. At admission, the child was alert, abnormal gait was evident, and a positive Romberg test was highlighted; no cranial nerve deficits were found. The brain computed tomography (CT) showed a hyperdense mass in the fourth ventricle with obstructive hydrocephalus (Figure 1). The magnetic resonance imaging (MRI) exam confirmed the presence of the heterogeneous intraventricular tumor with small hyperintense cystic components, heterogeneous contrast-enhancement, and reduced diffusion, suspicious for MB (Figure 1) without cerebral and spinal metastases. The lesion’s high density on the CT study and the reduced diffusion on MRI indicated high cellular density and nuclear area. Fullness at the level of the right foramen of Luschka suggested some laterality, a particular MRI feature associated with MB belonging to the WNT subgroup [11].

The patient underwent endoscopic third ventriculostomy, and subsequently, a gross total resection was performed. No neoplastic cells were detected in the cerebrospinal fluid. 

**Figure 2:**
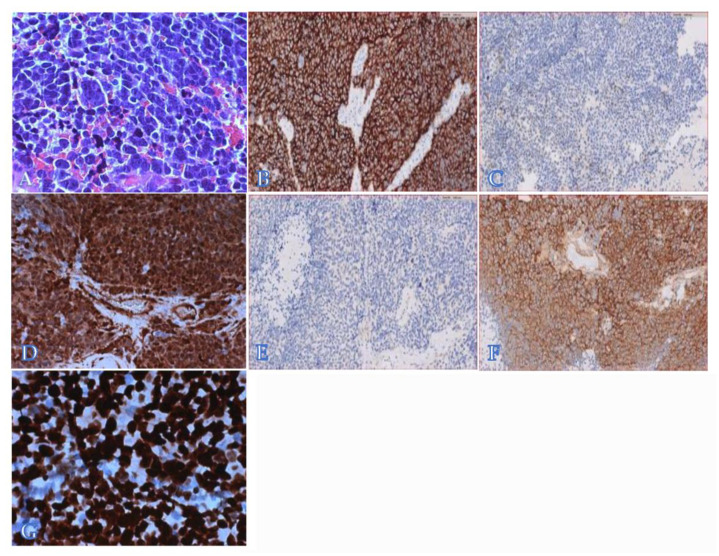
Histologically, the tumor consisted of sheath of cells with areas of anaplasia (**A**). The tumor showed diffuse expression of synaptophysin (**B**) and isolated NeuN-positive cells (**C**); YAP1 was diffusely positive (**D**), whereas GAB1 was negative (**E**). Beta-catenin expression was exclusively cytoplasmatic (**F**). TP53 was expressed by the majority of cells (**G**).

The histological examination revealed solid sheets of neoplastic cells characterized by high nuclear/cytoplasmic ratio and high mitotic index with focal necrosis. Areas with nuclear molding and cell wrapping were identified, consistent with focal anaplasia (Figure 2A).

The tumor was diffusely positive for synaptophysin (clone DAK-SYNAP, prediluted, high pH, Dako-Agilent) [Figure 2B], with isolated Neu-N-positive neoplastic cells (clone A-60, 1/100, low pH, Millipore) (Figure 2C). The YAP1 protein (clone 63.7, 1/100, high pH, Santa Cruz) was diffusely expressed (Figure 2D), whereas GAB1 (clone H-7, 1/100, high pH, Santa Cruz) was not detected (Figure 2E). Despite the absence of β-catenin nuclear expression (clone 17c2, 1/100, high pH, Leica) (Figure 2F), a pathogenic variant in the exon 3 of the corresponding *CTNNB1* gene was found by Sanger sequencing (c.98C>T; p.Ser33Phe). Overall, these data supported the diagnosis of MB, classic type, with focal anaplasia belonging to the WNT molecular group. Notably, the tumor was diffusely positive for *p53* (clone DO-7, prediluted, high pH, Dako-Agilent), suggesting a *TP53* variant (Figure 2G), as reported in a small fraction of patients (about 10%) of this molecular group [12]. However, no germline mutation of *TP53* was detected in this patient.

**Figure 3:**
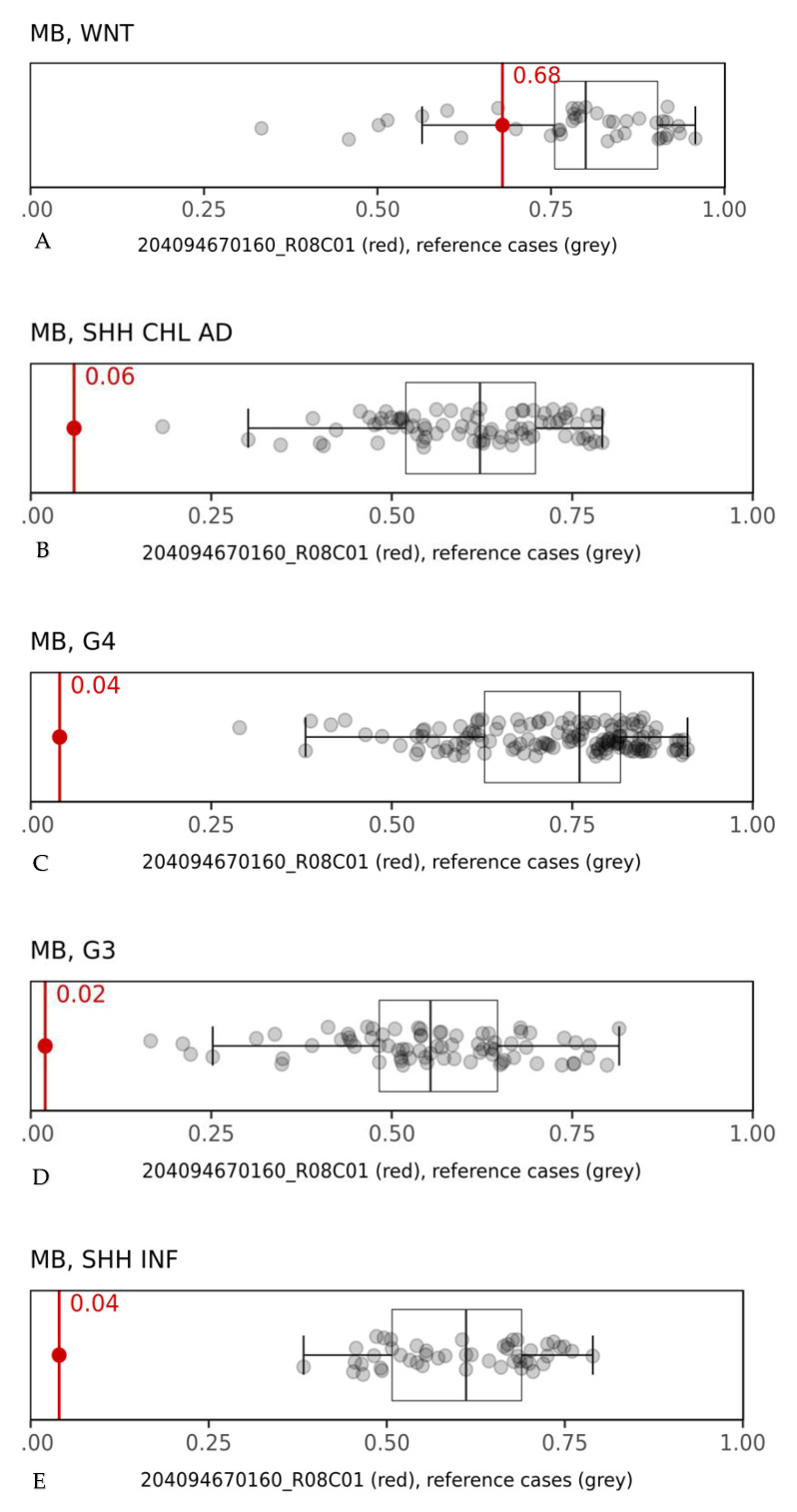
Box-and-whisker plots depicting the maximum raw classification scores (0.68) of the tumor sample in the methylation class “medulloblastoma, WNT” (**A**). Grey dots represent the reference cases in the various methylation class (**B**–**E**). MB: medulloblastoma.

DNA methylation profiling was performed on formalin-fixed and paraffin-embedded tissue (FFPE 250 ng) following protocols previously reported [13,14]. Protocols were approved by the Bambino Gesù Children’s Hospital Institutional Review Board, and written consent was obtained from the patient’s parents. The tumor had a raw classification score of 0.68 (Figure 3) corresponding to a calibrated score of 0.99 in the “methylation class medulloblastoma, WNT”, in line with the pathological findings [15]. 

**Figure 4:**
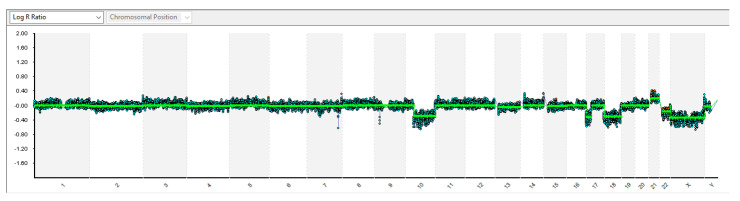
Single nucleotide polymorphism (SNP) array image by Bluefuse Multi Software: each chromosome, whose number is reported on the X axis, is represented as a grey or white area. Duplications/deletions are showed as an upward/downward shift of baseline (log R ratio = 0 for autosomal chromosomes). Low-level mosaicism (<20%) cannot be detected by this technique.

Single nucleotide polymorphism (SNP) and oligo-array analyses showed several imbalances in the DNA collected from tumor cells: homogeneous monosomy of chromosome 10q, 17p (where *TP53* gene maps), and of the whole chromosome 18 (Figure 4), as well as mosaic monosomy of chromosome 22 (about 40%). Evaluation of the B allele frequency also suggested the presence of a poorly represented cell clone (<20%) showing additional aneuploidies: trisomy 2, 4, 6, 7, 9, and 15, and tetrasomy 21. Neither monosomy of chromosome 6 nor amplification/gain of the *MYC*/*MYCN* genes were detected. To exclude the presence of mutations in high-risk cancer-predisposition genes, molecular genetic characterization by next-generation sequencing (NGS) using clinical exome sequencing (CES) (Twist Bioscience, San Francisco, CA, USA), including medulloblastoma predisposition genes (*APC*, *BRCA2*, *PALB2*, *PTCH1*, *SUFU*, *TP53*, and *GPR161*) were performed on genomic DNA extracted from circulating leukocytes of the patient and unaffected parents. Sequence analysis of DNA excluded the presence of germline variants in cancer predisposition genes. However, high-coverage sequencing on DNA extracted from the patient’s tumor, besides confirming exon 3 CTNNB1 gene mutation (NM_001904.3: c.98C>T; p.Ser33Phe), showed the presence of additional pathogenic somatic variants (Table 1). 

In line with p53 immunohistochemical overexpression and with the monosomy of 17p, a homozygous missense variant in TP53 gene, NM_000546.5: c.743G>A (p.Arg248Gln), was identified. Overall, our data indicated the occurrence of a somatic p53 mutation with loss of heterozygosity of the wild-type allele. Furthermore, the analysis revealed a monoallelic somatic variant in the *PIK3CA* gene, NM_006218.3: c.113G>A (p.Arg38His). The missense variant has been reported as an oncogenic mutation in the adaptor-binding domain (ABD) of the protein. Pathogenic variants in the ABD have a critical role in the disruption of the interaction between the ABD and kinase domains, thereby promoting an alteration in the conformation of the kinase domain that affects the enzymatic activity [16]. A somatic variant in the *SMARCA4* gene, c.3727C>T (p.Arg1243Trp), involving one of four hotspot mutations in the helicase domain of the gene was also detected. Almost 60% of *SMARCA4* alterations are missense mutations [17]. The gene is involved in the regulation of transcription by modulating chromatin accessibility [18], and the functional consequence of *SMARCA4* variants is crucial to identifying therapeutic strategies against tumors. Finally, a variant in the *FBXW7* gene, likely pathogenic, was found. This missense mutation, NM_018315.4: c.1154G>A (p.Arg385His), has been reported in several types of cancer [19]. 

Furthermore, data analysis revealed two known pathogenic compound heterozygous variants in the *DUOX2* gene, NM_014080.4: c.602dupG (p.Gln202ThrfsTer99) and c.1126C>T (p.Arg376Trp), inherited from the mother and the father, respectively. Biallelic loss-of-function mutations of DUOX2 observed in CH were found in our patient [20].

The patient was treated with a combination of surgery, radiotherapy, and chemotherapy. Unfortunately, the patient early relapsed with local and metastatic disease 2 months after the end of treatment, and the patient died 11 months after diagnosis.

## 3. Discussion

Multiple variants in genes on both chromosome 21 and at other sites in the genome contribute to the variation in clinical manifestations of DS, such as polymorphisms of the *DSCAM* (DS cell-adhesion molecule) and *APP* (amyloid precursor protein) genes [21]. DS is considered as a CPS, according to a higher rate of whole and segmental chromosomal instability, increased DNA damage and defective DNA repair, immunodeficiency and susceptibility to infections, and oncogenes on extra-copy of chromosome 21, including upregulation of pro-apoptotic and angiogenesis genes [22,23]. Peculiarly, the cancer distribution in DS differs from the one observed in the general population: neural malignancies, such as neuroblastoma, MB, and central nervous system primitive neuroectodermal tumors (CNS-PNETs), have a decreased incidence in the DS population [24,25,26], in contrast from other CPSs linked to an increased risk of MB (i.e., Gorlin syndrome (associated with mutations in *SUFU* and *PTCH1*), Li-Fraumeni syndrome (*TP53* mutations), *APC*-associated polyposis conditions, and Fanconi anemia (*BRCA2* mutations)) [7]. Only isolated cases of MB and CNS-PNETs have been described in individuals with DS [9,10]. In the largest epidemiological study on CNS pediatric tumors from a brain tumor registry of 13 countries, among a total of 6882 MBs and 1161 CNS-PNETs, only one patient with DS and MB was reported [26]. In contrast, children with DS represent 2% of all pediatric cases of acute lymphoblastic leukemia and 10% of pediatric cases of acute myeloid leukemia [2,21], with poorer outcomes than the ones observed in the general population [27,28]. 

The protective role of DS for the occurrence of solid tumors in general and in CNS is not clear. Nevertheless, few data have focused on the biological mechanism of the low incidence of MB in DS. As demonstrated in the Ts65Dn mouse model and confirmed on autopsy findings of DS and non-DS patients, the population with DS has cerebellar hypoplasia and a reduced density (up to less than 70%) of cerebellar granule neuron progenitors (CGNPs), the cells considered to be at the origin of MB [29]. Furthermore, the reduction of cerebellar volume in DS patients, evident when compared to age, gender, race-matched non-DS control individuals, has also been confirmed by MRI studies [10]. Additionally, supernumerary chromosome 21 appears to inhibit neuroectodermal differentiation of pluripotent embryonic stem cells [23] and is associated with an overexpression of the S-100 b protein [24]. The latter is thought, at least in part, to be responsible for the unusual rarity of neuroblastomas in DS, as it induces differentiation in neural cells. It could therefore be hypothesized that the genetic condition associated with DS has a protective effect against neural (non-glial) neoplasms, possibly also having a preventive role in the appearance of MB. 

In 2010, the Medulloblastoma Working Group identified four principal transcriptional subgroups of MB termed WNT, Sonic hedgehog (SHH), Group 3, and Group 4 [30], each with distinct phenotype traits, associated mutations, tumor cell histology, and patient prognosis. 

The WNT sub-group is the smallest, representing about 10% of all MBs, with an overall survival exceeding 90% on the current therapy [31,32]. These tumors are typically quite uniform in terms of genetic aberrations, histologic pattern, and clinical presentation. They commonly harbor pathogenic variants in the *CTNNB1* gene and monosomy of chromosome 6. Otherwise, WNT tumors harbor remarkably few genomic alterations. In addition, this subgroup of MBs is typically characterized by a remarkably good prognosis even when associated with unfavorable conditions such as CPS (constitutional *APC*-mutation) and *TP53* somatic mutation [7,33,34,35,36]. The diagnosis of WNT tumors can be established by several methods, with the most accurate being sequencing exon 3 of *CTNNB1*, DNA methylation profiling, or gene expression profiling, but a combination of immunohistochemistry for nuclear beta-catenin, pathologically expressed in alterations of WNT pathway, and fluorescent hybridization in situ or DNA copy number array profiling demonstrating monosomy 6 can also be used to reliably identify WNT tumors [12]. WNT-subgroup MBs originate from cells derived from the lower rhombic lip [11,37]. Their location and development occur along a triangle centered on the foramen of Luschka with one peak extending ventrolaterally to the cerebellopontine angle cistern, another postero-infero-medially to the cisterna magna, and the third postero-supero-medially to the IV ventricle [11]. This particular distribution led to the hypothesis that MBs of the WNT subgroup are close to the midline but are lateralized [38], as occurred in our clinical case, even though such lateralization, if subtle, cannot always be detected by imaging examinations. 

The presence of an extra copy of chromosome 21 has been related to an upregulation of pro-apoptotic and angiogenesis genes associated with premature aging processes, such as the dysregulation of the NOTCH/WNT pathway [22], which is probably linked to an increased risk of leukemia in the DS population [22]. Intriguingly, our patient’s methylation profile allocated his tumor to the WNT subgroup, suggesting that the deregulation of the WNT pathway may have played a critical role in the etiology of MB in our case, explaining a new possible pathogenetic mechanism.

This finding in a DS individual constitutes an unusual event, worthy of further study in a large MB series with broad molecular characterization, in order to elucidate the molecular mechanisms underlying this rare tumor in DS with particular regard to the role of the WNT pathway in this rare association. Moreover, in our case, the poor outcome of the patient was unusual for the WNT molecular group, also considering the presence of focal anaplasia [31,32,33]. Therefore, more studies with molecular characterizations and evaluation of long-term outcome would lead to more information about the impact of different molecular subtypes of MB in DS prognosis.

Finally, it is important to comment on the finding of a *TP53* somatic missense variant, in our case, associated with monosomy of chromosome 17p. Thus, our patient’s MB was characterized by the presence of the only pathogenetic *TP53* variant, while losing the second copy due to 17p monosomy. The variant found in our patient involves one of the most frequent somatic substitution hotspots in the *TP53* gene [39] and has been previously reported to be frequently associated with gain-of-function properties, being able to confer to the tumor a more aggressive phenotype [40,41]. A *TP53* pathogenic variant, when occurring in SHH MB, has a remarkable impact on patient outcome, defining a high-risk subgroup within the SHH MB group, which has a dismal prognosis. Although Tabori et al. [42] stated that somatic *TP53* mutation is always associated with a poor prognosis, several studies have reported a very good prognosis in the WNT MBs subgroup, even in the presence of *TP53* mutations [33,34,35]. One study showed that the activation of nuclear β-catenin can abrogate the radioresistance conferred by *TP53*-mutated MBs as a potential explanation for this difference in outcome [33]. Nevertheless, it is necessary to emphasize the extreme rarity of *TP53* mutation in patients with WNT MBs and the need to investigate this association, also in prognostic terms [33]. 

## 4. Conclusions

Our case is the first case of a WNT subgroup MB occurring in an individual with DS and the third case with molecular characterization (Table 2) [9,10]. The other two published cases belonged to Group 3 and to the SHH subgroup [10]. The latter occurred in a patient with Gorlin syndrome, a well-known predisposition syndrome for MB, suggesting that the MB pathogenesis was related to the *PTCH1* variant rather than to DS.

The nearly seven-fold decreased risk of MB in children with DS suggests the presence of a protective biological mechanism [25] and, as suggested by Satgé and Rickert, represents a medical enigma [43]. The cerebellum hypoplasia and the reduced volume of CGNPs seem to be potentially favorable “anatomic” conditions to preserve MB development, as well as the inhibition of neuroectodermal differentiation, which is instead observed in neuroblastoma. Moreover, the potential involvement of the WNT pathway should be further investigated in such cases. Given the poor outcome that is unusual for WNT molecular subgroup patients, it would be useful to also consider the association with other somatic mutations or specific histological findings (such as anaplasia) in this subgroup. 

Further evidence is needed to determine the protective role of DS in terms of CNS tumors and MB occurrence through larger series with molecular characterizations that could clarify the related biological mechanism(s). 

## Figures and Tables

**Table 1:** Prediction analysis and American College of Medical Genetics (ACMG) classification of somatic variants observed with next-generation sequencing (NGS) analysis of tumor tissue.

GENE	RefSeq	HGVS	DBSNP	ACMG	Location
*TP53*	NM_000546.5	c.743G>A p.Arg248Gln	rs11540652	Pathogenic	17:7577538
*CTNNB1*	NM_003072.3	c.3727C>T p.Arg1243Trp	rs121913400	Likely pathogenic	3:41266101
*SMARCA4*	NM_001024847.2	c.118G>A p.Asp40Asn		Likely pathogenic	19:11144146
*FBXW7*	NM_018315.4	c.1154G>A p.Arg385His	rs1057519895	Likely pathogenic	4:153249384

**Table 2:** Medulloblastoma in the Down syndrome (DS) population in literature.

Reference (Number of Patients)	Presence of Another CPS	Age	Histological Classification	Molecular Finding	Outcome
Benesch M. et al., Pediatr Blood Cancer, 2009 (9) [1]	No	4 years	Medulloblastoma	Group 3 (personal communication: Capper et al.: 2015 [6])	Alive at 60 months in complete remission
Mangum R. et al., Childs Nerv Syst, 2016 (10) [1]	Yes (Gorlin Syndrome)	21 months	Medulloblastoma desmoplastic/nodular Synaptofisin Neu-N: positive GFAP (Glial fibrillary acidic protein), Neurofilament proteins: negative β-catenin: negative N-myc/C-myc amplification: negative	SHH subgroup Heterozygous *PTCH1* variant (c.834G>A) predicted to result in premature protein termination (p.Trp278 *)	Not available
Our case (1)	No	10 years	Classic Medulloblastoma with focal anaplasia grade IV (according to WHO 2016) Synaptofisina, Neu-N: positive YAP1: diffusely expressed GAB1: negative β-catenin negative (but a pathogenic *CTNNB1* variant in exon 3) p53: positive N-myc/C-myc amplification: negative	WNT subgroup	Died at 11 months from diagnosis

## Abbreviations

DS	Down syndrome
MB	medulloblastoma
WNT	wingless
CNS	central nervous system
CPS	cancer predisposition syndrome
CT	computed tomography
MRI	magnetic resonance imaging
SNP	single nucleotide polymorphism
NGS	next-generation sequencing
CES	clinical exome sequencing
CH	congenital hypothyroidism
ABD	adaptor-binding domain
CNS-PNET	central nervous system primitive neuroectodermal tumors
SHH	Sonic hedgehog
CGNPs	cerebellar granule neuron progenitor cells
